# ICON: Eosinophil Disorders

**DOI:** 10.1097/WOX.0b013e31827f4192

**Published:** 2012-12-15

**Authors:** Peter Valent, Amy D Klion, Lanny J Rosenwasser, Michel Arock, Bruce S Bochner, Joseph H Butterfield, Jason Gotlib, Torsten Haferlach, Andrzej Hellmann, Hans-Peter Horny, Kristin M Leiferman, Georgia Metzgeroth, Kenji Matsumoto, Andreas Reiter, Florence Roufosse, Marc E Rothenberg, Hans-Uwe Simon, Karl Sotlar, Peter Vandenberghe, Peter F Weller, Gerald J Gleich

**Affiliations:** 1 Department of Medicine I, Division of Hematology & Hemostaseology, Medical University of Vienna, Austria, Wahringer Gurtel 18-20, Wien A-1090 Austria; 2 Eosinophil Pathology Unit, Laboratory of Parasitic Diseases, NIH/NIAID, Bethesda, MD; 3 Children's Mercy Hospital, Kansas City, MO; 4 LBPA CNRS UMR8113, Ecole Normale Supérieure de Cachan, Cachan, France; 5 Department of Medicine, Division of Allergy and Clinical Immunology, Johns Hopkins University School of Medicine, Baltimore, MD; 6 Division of Allergic Diseases, Mayo Clinic, Rochester, MN; 7 Division of Hematology, Stanford Cancer Center, Stanford, CA; 8 MLL Münchner Leukämielabor, Munich, Germany; 9 Department of Hematology, Medical University School of Gdansk, Gdańsk, Poland; 10 Institute of Pathology, Ludwig-Maximilians-University, Munich, Germany; 11 Department of Dermatology, University of Utah Health Sciences Center, Salt Lake City, UT; 12 III. Medizinische Klinik, Universitätsmedizin Mannheim, Universität Heidelberg, Mannheim, Germany; 13 Department of Allergy and Immunology, National Research Institute for Children's Health and Development, Tokyo, Japan; 14 Department of Internal Medicine, Erasme Hospital, Université Libre de Bruxelles, Brussels, Belgium; 15 Division of Allergy and Immunology, Cincinnati Children's Hospital, Medical Center, Cincinnati, OH; 16 Institute of Pharmacology, University of Bern, Bern, Switzerland; 17 Center for Human Genetics, University Hospitals Leuven and Katholieke Universiteit Leuven, Leuven, Belgium; 18 Department of Medicine, Beth Israel Deaconess Medical Center, Harvard Medical School, Boston, MA; 19 Department of Medicine, University of Utah Health Sciences Center, Salt Lake City, UT

**Keywords:** eosinophil disorders, hypereosinophilic syndrome (HES), global consensus, classification

## Summary

Eosinophil disorders and related syndromes are a heterogeneous group of conditions characterized by marked persistent blood eosinophilia and involvement of one or more organ systems. The hypereosinophilic (HE) state is defined by a persistent eosinophil count exceeding 1.5 × 10^9^/L blood. Several different neoplastic, paraneoplastic, infectious, and allergic disorders may underlie HE. Eosinophil-induced organ damage with more or less typical symptoms may develop in these patients. The final diagnosis is based on clinical, molecular, and histopathologic criteria, and the presence of signs and symptoms indicative of HE-induced organ damage, the latter often manifesting as hypereosinophilic syndrome. The clinical course, prognosis, and response to certain drugs vary greatly among patients and among disease variants. During the last few years, several new markers and targets have been identified, improving diagnosis, prognostication, and therapy for patients with HE-related disorders. Moreover, several attempts have been made to establish robust disease-related criteria and a global classification for HE-related diseases. However, the pathogenesis and mechanisms of HE and of HE-induced organ damage are complex, and expert opinions remain divided.

In light of the increasing burden of allergic diseases, the World Allergy Organization; the American Academy of Allergy, Asthma & Immunology; the European Academy of Allergy and Clinical Immunology; and the American College of Allergy, Asthma & Immunology have come together to increase the communication of information about allergies and asthma at a global level. Within the framework of this collaboration, termed the International Collaboration in Asthma, Allergy and Immunology, a series of consensus documents called International Consensus ON (ICON) are being developed to serve as an important resource and support physicians in managing different allergic diseases.

This ICON provides an updated proposal for a global nomenclature and classification of HE-related disorders and conditions. The proposal is based on the currently available literature and merges previously published proposals and the classification proposal of the World Health Organization.

The International Collaboration in Asthma and Allergy initiated an international coalition among the American Academy of Allergy, Asthma & Immunology; European Academy of Allergy and Clinical Immunology; World Allergy Organization; and American College of Allergy, Asthma and Immunology on eosinophilic disorders. An author group was formed and then divided into individual committees. Within the committee, teams of authors were created to generate content for specific sections of the article. Content was derived from literature searches, relevant published guidelines, and clinical experience. After a draft of the document was assembled, it was collectively reviewed and revised by the authors. Where evidence was lacking or conflicting, the information presented represents the consensus expert opinion of the group.

## Proposed Nomenclature and Review of the Literature

The published literature was screened for terminologies, nomenclatures, criteria, classification proposals, and key information concerning incidence, prevalence, and prognostication of eosinophil disorders. Key publications are referenced. Whenever found, controversies and difficulties in nomenclatures and classifications were reviewed and discussed. In this way, the current International Consensus ON (ICON) article attempts to adhere to the principles of previous (traditional) classifications, including World Health Organization (WHO) criteria for eosinophilia-associated hematopoietic neoplasms. In addition, a recent proposal of the "International Cooperative Working Group on Eosinophil Disorders" (ICOG-EO) was used as the basis for the formulation of the ICON document. The ICOG-EO was established in 2011 as an interdisciplinary network, including representatives from the fields of allergy, immunology, pathology, hematology, infectious diseases, and molecular medicine.

Traditionally, the hypereosinophilic syndrome (HES) has been described as a condition associated with marked persistent peripheral blood eosinophilia, organ damage, and exclusion of an underlying disease or condition that could otherwise explain eosinophilia or organ damage [1-5]. The plural form (ie, HES) was subsequently proposed to reflect the extremely heterogeneous conditions ultimately resulting in eosinophil-mediated organ involvement. A rare familial type of HES has also been described. As per the ICOG-EO, HES has been redefined as any form of HE (not just idiopathic) associated with organ damage. Thus, HESs can be divided into primary (neoplastic) HES, secondary (reactive) HES, and idiopathic HES [4-9]. The ICOG-EO group recommends that the term HES be reserved for such clinical syndromes but not for underlying malignancy or autoimmune disease associated with eosinophilia [9]. In primary (neoplastic) HES or secondary HES, an underlying disease is identified and is included in the final diagnosis, for example, chronic eosinophilic leukemia (CEL) with primary HES. In contrast, a diagnosis of idiopathic HES implies the absence of a known etiology.

Using this nosology, the term HES should be applied to document and describe syndromes with eosinophil-induced organ damage, irrespective of the presence of an underlying etiology. In fact, the diagnosis of HES should prompt physicians to investigate and search for underlying mechanisms and disorders [8,9]. In previous WHO classifications, HES was sometimes employed as a synonym of CEL and sometimes to discriminate CEL from other hematologic conditions/neoplasms [10-13]. In the latest edition of the "WHO monograph, "[12] the term "HES" is not recommended as a synonym of WHO-defined neoplasms. This distinction between HES and the underlying disorder is in agreement with the proposal of the ICON group [9].

**Figure 1:**
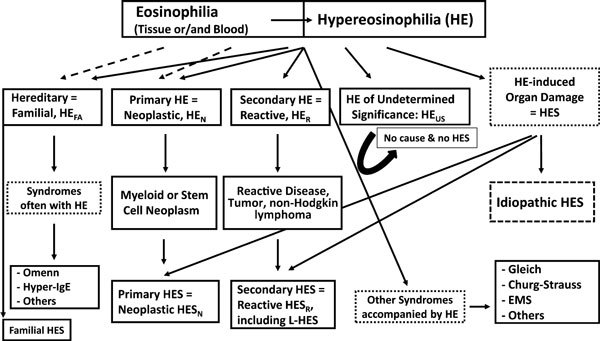
**Diagnostic Algorithm in patients with Eosinophilia**. In a first step, the presence of eosinophilia or hypereosinophilia (HE) is confirmed. Then, the etiology of eosinophilia/HE is studied, and the patient is examined for the presence of HE-related organ damage. Based on results, patients are classified into various hematologic or immunologic diseases. In those who are suffering from HE-related organopathy, the additional diagnosis of hypereosinophilic syndrome (HES) is established. In a small proportion of cases, familial HES or rare syndromes presenting with HE, such as the Gleich syndrome or Churg Strauss syndrome (CSS), are diagnosed. L-HES, lymphoid variant HES; EMS, eosinophilia myalgia syndrome.

The spectrum of nonneoplastic and neoplastic disorders that can underlie HES is broad, and allergic disorders are often important considerations in the differential diagnosis [14-23]. Molecular, immunological, and histopathological signs of clonality of myeloid cells (eosinophils) as well as clinical and laboratory features suggesting the presence of a reactive process should be looked for in these patients [14-18]. A diagnostic algorithm is shown in Figure 1. Among nonneoplastic (reactive) disorders, common conditions such as helminth infections or allergic diseases but also rare conditions and syndromes have to be considered (Table 1) [19-23].

**Table 1:** Conditions associated with Hypereosinophilia (HE)

Reactive conditions
Helminth infections
Allergic reactions
Atopic diseases
Drug reactions (allergic or toxic)
Scabies, other infestations
Allergic bronchopulmonary aspergillosis
Autoimmune diseases
Other chronic inflammatory diseases
Chronic graft-vs-host disease
Lymphocytic/lymphoid variant HES
Neoplastic conditions involving the hematopoietic system (see Table 2)
Myeloid neoplasms
Mast cell neoplasms
Lymphoid neoplasms
Paraneoplastic conditions
Solid tumors/malignancy
Lymphoproliferative neoplasms
Idiopathic forms
Idiopathic eosinophilia
HE of uncertain (undetermined) significance
Idiopathic HES
Rare syndromes associated with HE
Gleich syndrome
CSS
EMS
Omenn syndrome
Hyper IgE syndrome
Hereditary HE (not otherwise specified)

CSS, Churg-Strauss Syndrome; EMS, Eosinophilia Myalgia Syndrome.

It is noteworthy that blood hypereosinophilia (HE) is not necessarily accompanied by organ damage (ie, criteria for HES not fulfilled), even in the presence of an underlying disorder typically presenting with chronic HE [9]. This is especially true when HE is detected early in the disease process.

When a persistent (hyper)eosinophilia is demonstrated in the absence of organ involvement or an underlying disease, the (provisional) diagnosis of HE of uncertain significance is established [9]. Table 1 shows a summary of conditions associated with HE.

## Definition and Classification of HE-Related Conditions and Disorders

The diagnostic algorithm should start at the important checkpoint of peripheral blood HE, defined as a persistent elevation of blood eosinophils above 1.5 × 10^9^/L blood [5-9]. The term "tissue HE" has also been proposed and may be useful in the evaluation and classification of HE-related disorders [9]. However, the clinical impact of isolated tissue HE (without blood HE) is difficult to determine because robust data from large studies are unavailable. In addition, documentation of tissue HE sometimes requires special stains directed against eosinophil granule proteins. Therefore, blood HE is considered the most important primary denominator and checkpoint when approaching HE-related disorders [1-13]. In some patients, criteria for blood HE are not yet met, but molecular and clinical signs are strongly indicative of a particular eosinophilic disorder with or without an accompanying HES. These patients should be followed closely because they may progress to overt eosinophilic disease over time.

At the checkpoint HE, 2 critical questions have to be answered to make a final diagnosis: (1) is there an underlying disease or condition and (2) are there clinical signs and symptoms or laboratory abnormalities that point to the presence of accompanying HE-induced organ damage (HES)[9] (Figure 1). For example, the hematologic work up reveals eosinophilic leukemia, and the staging investigations show endomyocardial thrombosis/fibrosis. The final diagnosis in this patient is CEL with primary HES. In such patients, with HE and clinical manifestations that are typical of HES, histopathology is rarely required for diagnosis. However, in cases of rare or/and atypical organ manifestations (such as renal failure or bloody diarrhea), tissue biopsy may be required to document tissue HE and HE-induced organ damage, to establish the diagnosis of HES. The demonstration of extensive extracellular deposition of eosinophil-derived proteins, for example, eosinophil major basic protein (MBP) supports the conclusion that the organopathy is "HE-related."[24-27]

In patients with documented HE, 4 important groups of underlying disorders (conditions) can be identified: (1) hematopoietic neoplasms, (2) other (nonhematopoietic) neoplasms (paraneoplastic HE), (3) common allergic, reactive, or immunologic conditions, and (4) rare clinically defined syndromes accompanied by HE, including rare inherited disorders.

### Hematologic Neoplasms

Although many different hematologic disorders (neoplasms) can be accompanied by eosinophilia (Table 2), only a few are consistently accompanied by clonal neoplastic (primary) HE (HE_N_), and only very few neoplasms present with HE plus HE-related organ damage, that is HES (HES_N_). Myeloid neoplasms accompanied by HE (HE_N_) include the rare variant of acute eosinophilic leukemia, the more common chronic form of eosinophilic leukemia (CEL) that is often associated with the *FIP1L1-PDGFRA *rearrangement and endomyocardial thrombosis/fibrosis (HES_N_), and other myeloid neoplasms with rearrangements involving *PDGFRA, PDGFRB*, or *FGFR1*, such as the 8p11 syndrome [8-18,28-30]. HE_N _may also accompany untreated Ph+ chronic myeloid leukemia (Table 2). Rarely, HE_N _is found in patients with myelodysplastic syndromes (MDS) [31]. In most cases with dysplastic marrow and HE, additional signs of myeloproliferation are found, consistent with a diagnosis of MDS/myeloproliferative neoplasm (MPN) overlap disease or frank MPN [8-13]. This is also true for cases of HEN exhibiting either a *PDGFR *or *FGFR1 *rearrangement. In other variants of MPN, MDS, or MPN/MDS, eosinophilia is less commonly detected. Another myeloid neoplasm that is often accompanied by HE is systemic mastocytosis (SM)[32-34] (Table 2). Clonal eosinophilia is frequently seen in advanced stages of SM, that is. aggressive SM and mast cell leukemia, and many of these patients develop HE (usually without HES) [33].

**Table 2:** Hematopoietic Neoplasms Accompanied by Eosinophilia

Neoplasms in which eosinophils are likely to be clonal cells
Acute eosinophilic leukemia
CEL
Acute myeloid leukemia with inv(16) (FAB AML M4eo)
Chronic myeloid leukemia
Myeloid neoplasms with *PDGFR *abnormalities (WHO types)
Hematopoietic neoplasms with *FGFR1 *abnormalities (WHO types)
Smoldering systemic mastocytosis
Aggressive systemic mastocytosis
Mast cell leukemia
Neoplasms in which eosinophils may or may not be part of the malignant clone
Other MPN with eosinophilia*
MDS with eosinophilia
Other MDS/MPN overlap syndromes with eosinophilia*
Indolent systemic mastocytosis
Neoplasms in which eosinophils usually are not part of the malignant clone
Hodgkin disease
B-or T-cell non-Hodgkin lymphoma
Acute lymphoblastic leukemia
Chronic lymphocytic leukemia
Langerhans cell histiocytosis

*Other MPN or MPN/MDS: neoplasms where no abnormalities in the *PDGFR *or *FGFR1 *genes are detectable.

CEL, chronic eosinophilic leukemia; PDGFR, platelet-derived growth factor receptor; FGFR, fibroblast growth factor receptor; MPN, myeloproliferative neoplasm (s); MDS, myelodysplastic syndrome(s).

WHO, World Health Organization.

Lymphoid neoplasms can also present with HE. In most cases, T-cell lymphoma is diagnosed, and eosinophils are nonclonal cells [35-37]. However, in patients with the 8p11 syndrome and a few other rare entities, both eosinophils and lymphocytes can be involved in the clonal neoplastic process [16,38].

### Paraneoplastic Conditions Associated With HE

Several different types of cancers can be accompanied by (or even preceded by) eosinophilia [39]. In some patients, overt HE or even HES may develop. Cancers associated with HE include adenocarcinomas of the lung, gastrointestinal (GI) tract, pancreas, and thyroid, gynecological tumors, and skin cancers [39]. Although the pathogenesis remains unclear, a commonly accepted hypothesis is that cancer cells or the cancer-related microenvironment (eg, fibroblasts) produce eosinophilopoietic cytokines.

### Common Reactive/Immunologic Conditions

A number of different immunologic and other reactive conditions can cause reactive HE (HE_R_). These include infectious diseases (eg, helminth infections, HIV, and certain fungal infections), allergic disorders (eg, allergic asthma, food allergy, atopic dermatitis, and drug reactions, including the DRESS syndrome), and chronic inflammatory/autoimmune disorders [6,19,20,22,23,40]. Although not routinely tested, increased production of eosinophilopoietic cytokines in vitro by clonal T lymphocytes and/or lymphocytes with an aberrant surface phenotype can be documented in some cases of HE_R _in the absence of a T-cell lymphoma [41-43]. In such cases, HE_R_-related organ damage is often seen, confirming the diagnosis of the lymphoid variant of HES (L-HES) [4,37].

### Rare Syndromes Accompanied by HE

These include, among others, episodic angioedema and eosinophilia (Gleich syndrome), Churg-Strauss syndrome (CSS), Eosinophilia Myalgia syndrome (EMS), Omenn syndrome, and the Hyper-IgE syndrome (Table 1) [44-51]. Gleich syndrome is a disease characterized by recurrent angioedema associated with blood eosinophilia and elevated serum IL-5 and IgM levels (polyclonal IgM) [4,44,45]. In some of these patients, lymphocyte phenotyping reveals an abnormal (activated) T-cell compartment (CD4^+ ^T cells with decreased or absent membrane expression of CD3). Based on this observation, Gleich syndrome has also been regarded as a special subvariant of L-HES. Typical features in patients with CSS are a necrotizing vasculitis accompanied by asthma and eosinophilia [46-48]. In a subgroup of these patients, antineutrophil cytoplasmic antibodies are detectable. EMS is a condition defined by myalgia and eosinophilia, often accompanied by neurological symptoms and skin abnormalities [49-51]. Epidemic cases of EMS have been reported in the context of exposure to contaminated L-tryptophan or rapeseed oil (toxic oil syndrome). Omenn syndrome and Hyper-IgE syndrome are rare inherited immunodeficiency syndromes presenting with eosinophilia. There are also other clinical conditions and syndromes that may present with HE and resemble HES. In many of these conditions, eosinophilia is triggered by aberrantly produced eosinophilopoietic growth factors, such as IL-5 or GM-CSF.

## Prevalence and Epidemiologic Features

To date, little is known about the incidence and prevalence of HE and HES or the frequency and incidence of neoplastic and nonneoplastic conditions underlying HE and HES [52]. This is due to the rarity of these conditions, poor general knowledge about disease variants, and recent technological advances that have led to the identification of several (potentially) underlying pathogenic mechanisms, clinical correlates, and drug targets during the last 10 years. The epidemiological features of HE and HES are also poorly understood. Some eosinophil-related neoplasms show a clear gender prevalence, such as PDGFRA-rearranged CEL, which afflicts males in the vast majority of cases, [53] whereas in other leukemias accompanied by eosinophilia, no obvious gender predominance has been described.

## Pathophysiology

The degree and pattern of organ involvement in eosinophilic disorders are governed by 2 distinct factors. The first is the increased production and/or persistent accumulation of (normal or neoplastic) eosinophils that leads to HE and predisposes to or triggers the development of HES (high effector cell burden). The second factor is (persistent) "activation of eosinophils, " which is responsible for the clinical manifestations of HES. In many instances, both eosinophilia and eosinophil activation are present and have similar (or the same) underlying causes (eg, eosinophil-targeting cytokines). In other cases, HE may persist for many years without evidence of eosinophil activation or clinical manifestations [9].

Several basic mechanisms can lead to the persistent accumulation of eosinophils in blood and tissues, including increased proliferation and/or survival of eosinophil-committed progenitor cells, and enhanced survival of mature eosinophils. In principle, 2 pathogenetically different conditions can trigger eosinophil growth and accumulation, namely (1) an intrinsic defect of eosinophil-committed neoplastic progenitor cells, caused by mutations including those involving *PDGFR *or *FGFR1*, and (2) overproduction of cytokines, such as IL-3 or IL-5, that stimulate the growth, differentiation, and survival of (normal or neoplastic) eosinophils and eosinophil precursor cells_._[40,54,55] Although in most cases, only one of these two pathologic mechanisms may contribute to the development of HE, there are patients in whom neoplastic eosinophils have been described to produce autocrine eosinophil growth factors (IL-3, IL-5) themselves Furthermore, overproduction of HE-promoting cytokines may stem from another (noneosinophilic) clonal process such as a T-cell lymphoma or the L-HES [4,37].

As mentioned above, eosinophil activation is often triggered by the same or similar factors that induce HE. Likewise, most of the eosinophilopoietic cytokines, including IL-3 and IL-5, induce activation and tissue homing of mature blood eosinophils [56-58]. It is also well known that neoplastic eosinophils that derive from *PDGFR*-rearranged clonal precursor cells are in an "activated state"[59] and often cause HES-related organ damage. Nevertheless, not all patients with HE develop HES consistent with a multifactorial pathogenesis of end-organ damage in eosinophilic diseases.

Little is known about the role of eosinophil-derived mediators and molecules in the development of HES-related organ damage. Eosinophils are a rich source of various proinflammatory mediators and cytokines, including growth factors and chemotactic peptides and vasoactive, profibrotic, and angiogenic molecules [60-65]. In addition, eosinophils produce various lipid mediators and cytotoxic proteins, including eosinophil cationic protein (ECP), eosinophil major basic proteins (MBP1 and MBP2), eosinophil peroxidase (EPO), and eosinophil-derived neurotoxin (EDN) [64-67]. Eosinophil-derived peptide mediators and cytokines have diverse biological properties. Some of these proteins are directly toxic to cells and microorganisms, [65-67] whereas others act indirectly by activating various immune and nonimmune cells. Together, these mediators and substances, when released from activated eosinophils, can cause tissue remodeling and/or tissue damage. Eosinophil products can also activate platelets and endothelial cells and alter the production/expression of prothrombotic and antifibrinolytic substances, contributing to tissue fibrosis and thrombosis.

## Clinical Manifestations and Diagnosis

Depending on the type of disease and other factors, a number of different organ systems may be involved in patients with HES. The most commonly involved organs are the skin, lungs, GI tract, heart, and the central nervous system. In some patients, the relationship between the eosinophilia and typical clinical signs and symptoms are pathognomonic, whereas in others tissue biopsy may be required to document eosinophilia and tissue HE.

Endomyocardial thrombosis and fibrosis are often documented in primary (neoplastic) HES (HES_N_), particularly in association with the *FIP1L1-PDGFRA *fusion gene, but are also seen in other variants of HES [68-70]. A thorough cardiac evaluation including an electrocardiogram and echocardiogram is mandatory in all patients with HE. Cardiac magnetic resonance imaging is helpful in distinguishing eosinophilic cardiac disease from that of other etiologies. In some cases, troponin levels may also be helpful. An endomyocardial biopsy may be required in some cases for definitive diagnosis but is usually not performed. The presence of eosinophils in the myocardium is always abnormal and highly suggestive of eosinophil-mediated organopathy. In addition, the biopsy may reveal local thrombosis and fibrosis.

The respiratory system is commonly involved in HES [71,72]. To confirm that respiratory manifestations are related to HE, a number of studies can be helpful, including pulmonary function tests, chest radiography and/or computed tomography scan, and bronchoscopy with bronchoalveolar lavage and tissue biopsy if necessary. The differential diagnosis of HES_R _with pulmonary involvement includes drug allergy, anti-neutrophil cytoplasmic antibodies--negative CSS, chronic eosinophilic pneumonia, severe allergic bronchial asthma with eosinophilia, allergic bronchopulmonary aspergillosis, and other eosinophilic lung infections.

An important biomarker for asthma control and response to therapy is induced sputum eosinophilia. This finding has proven to be a useful and consistent marker of inflammation in asthma at all levels of severity and control [73,74].

The evaluation of skin involvement in HES is a clinical and histopathologic challenge [72,75]. Consequently, an experienced dermatologist should be involved in making the diagnosis of HES on the basis of skin involvement. HES may be associated with a large spectrum of dermatologic manifestations, including papules, eczema, angioedema, urticaria, erythrodermia, mucosal (oral and genital) ulcers, and necrotizing vasculitis. In some patients, skin lesions may be severe and debilitating despite therapy.

The bone marrow is frequently affected in HES. In idiopathic HES and HES_R_, there is usually a mild to moderate tissue eosinophilia without alteration of the bone marrow microarchitecture and without abnormalities in other hematopoietic lineages. In contrast, in HES_N_, there is often marked tissue eosinophilia leading to substantial hypercellularity in the bone marrow and alteration of its microarchitecture. Other hematopoietic lineages show typical aberrations according to the underlying hematopoietic neoplasm, which might be an MPN, MDS, MDS/MPN overlap disease, acute myeloid leukemia (AML), or, rarely, a lymphoid neoplasm (Table 2). In these patients, various blood count abnormalities may be present, such as thrombocytosis, monocytosis, basophilia, and a left shift or/and a slight increase in blast cells. In some cases, an advanced myeloid neoplasm may be detected. In other cases, anemia or/and thrombocytopenia with or without increased blast cells or dysplasia is found, consistent with the diagnosis of an MDS, MDS/MPN overlap disease, or (primary or secondary) AML. In rare cases, stem cell disease with involvement of the myeloid and lymphoid lineage is identified. In some of these patients, the 8p11 syndrome is diagnosed. The prognosis is poor in these patients.

GI tract involvement is common in HES, [72,76,77] particularly in patients with HES_R _or the idiopathic form of HES. In these patients, a wide variety of symptoms and findings have been described, including abdominal pain, vomiting, chronic diarrhea, or chronic ulcerative disease [72,77].

## Treatment Options

Patients with HE without symptoms may not require any therapy. However, in case of organ damage (HES), treatment has to be initiated. The type of therapy varies depending on the underlying condition and type of HES. Reactive HES is best managed by treating the underlying disease [78-80]. In patients with primary (neoplastic) HE or HES, specific therapy with targeted drugs or chemotherapy may be required. If the underlying disease is resistant, eosinophil counts can usually be kept under control with hydroxyurea or interferon alpha (IFN-α). For patients with idiopathic HES or L-HES, glucocorticoids are often prescribed [78-80]. However, chronic use of glucocorticoids is often associated with substantial side effects. Currently available corticosteroid-sparing agents that can be used in these patients include hydroxyurea and IFN-*α *[78-80]. Mepolizumab, a humanized anti-IL-5 antibody, has also been shown to be a safe and effective steroid-sparing agent in patients with non-neoplastic (steroid-responsive) HES, [81,82] and in patients with asthma and CSS. However, the drug is currently unavailable for use in clinical practice.

In contrast to patients with idiopathic HES and HES_R_, patients with HES_N _typically respond only partially or transiently to glucocorticoid therapy. IFN-α and hydroxyurea have been used with some success in patients with HES_N_.

However, in patients with rearranged *PDGFRA *or *PDGFRB*, imatinib is usually effective and is thus used as standard first-line therapy [83-86]. Based on the potent activity of this kinase blocker in such patients, it is essential to search for and define these molecular defects in all patients with suspected CEL or HES_N_.

The most frequent fusion oncoprotein and target detectable in patients with HES_N _(usually in CEL patients) is FIP1L1-PDGFRA [84-87]. This oncogenic gene product is a target of imatinib and is also sensitive to other tyrosine kinase inhibitors (TKIs), such as nilotinib or dasatinib. Other molecular targets of imatinib are also detectable in patients with HES_N _(including CEL), albeit at lower frequency. Most of these fusion genes involve *PDGFRA *or *PDGFRB*. By contrast, FGFR1fusion proteins are resistant both to imatinib and to most other TKIs.

For patients with FIP1L1-PDGFRA+ disease, imatinib is now considered standard first-line treatment. Most patients have a long-lasting response to this drug. The recommended starting dose is usually 100 to 400 mg daily, and in the majority of patients, remission is maintained with low-dose treatment (100 mg daily), although some patients may require 400 mg daily to keep the eosinophil counts under control [84-88]. Primary and secondary resistance to imatinib occurs, but is uncommon, and is generally caused by rare mutations in *FIP1L1-PDGFRA*. Newer TKIs, such as sorafenib, nilotinib, midostaurin, and ponatinib, are an option in the setting of drug resistance (as experimental agents, in clinical trials). Another approach is the use of alternative cytoreductive agents, such as IFN-α, hydroxyurea, or polychemotherapy. In patients who progress to AML or who have resistant disease with life-threatening clinical manifestations, high-dose chemotherapy and hematopoietic stem cell transplantation must be considered.

In most patients with FGFR1-derived fusion proteins, imatinib is ineffective and thus is not recommended [86,87]. In many of these patients, the condition behaves as an aggressive stem cell disease or a leukemia/lymphoma syndrome, also known as 8p11 syndrome. In these patients, chemotherapy with consecutive allogeneic stem cell transplantation is usually recommended. An alternative may be the use of novel more potent TKI, such as ponatinib.

## Unmet Needs

There are several unmet needs in the field of eosinophil disorders, not only in terms of basic and applied science and in the management of disease variants but also with respect to terminologies, classification, and prognostication. These issues should be addressed in a comprehensive and multidisciplinary way, which is the main goal of the recently established ICOG-EO working group. With regard to classifications, it is important to separate the clinical syndrome HES and HES-defining criteria from criteria and definitions used to establish the final diagnosis of an underlying neoplastic (hematologic) or nonneoplastic disease. A good example of a similar situation is lymphoma, where B symptoms (eg, weight loss or fever) may be recorded based on defined criteria, but these criteria are not used in the histopathologic diagnosis of the lymphoma. Thus, a given lymphoma may or may not present with B symptoms. Similarly, eosinophil disorders may or may not present with HE-related organ damage (HES). Another unmet need is to validate all new classification proposals, including the proposal of the ICOG-EO working group. Notably, several different new criteria, provided by the WHO, ICOG-EO, or other groups, are based primarily on expert opinion rather than on robust studies. There is of course published evidence supporting each of these newly proposed terms or criteria, but only robust global validation will lead to broad acceptance and proper application of the criteria in clinical practice. In this regard, large (global) registries and trials must be established, wherein even rare subcategories of eosinophil disorders can be analyzed in terms of their incidence, prevalence, disease course, prognosis, and treatment response.

## Competing interests

The authors declare that they have no competing interests.

## Note

Supported in part by the Division of Intramural Research, NIAID, NIH.

This article is a product on eosinophil disorders by the iCAALL, an international coalition between the World Allergy Organization; American Academy of Allergy, Asthma & Immunology; European Academy of Allergy and Clinical Immunology; and American College of Allergy, Asthma & Immunology.
